# Coherent Structure Formation through nonlinear interactions in 2D Magnetohydrodynamic Turbulence

**DOI:** 10.1038/s41598-017-13943-7

**Published:** 2017-10-23

**Authors:** Elisa De Giorgio, Sergio Servidio, Pierluigi Veltri

**Affiliations:** University of Calabria, Department of Physics, Rende, I-87036 Italy

## Abstract

Using high resolution 2D magnetohydrodynamic (MHD) simulations we analyze the formation of coherent structures induced by nonlinear interactions in turbulent flows. The properties of these coherent structures, which at the smallest scales are identified through a spatial intermittent behavior, turn out to be guided by the conservation of ideal quadratic (rugged) invariants of the 2D incompressible MHD equations. Different spatial regions can be identified, where the correlations predicted using the variational principles associated to the rugged invariants are locally displayed. These local correlated structures are produced rapidly, as soon as the turbulence is fully developed. It is worth speculating that the small scale structures under our investigation could give rise to singular weak solutions when letting the dissipative coefficients go to zero. In this case their properties could furnish a key to understand which mathematical conditions characterize singularity emergency in weak solutions of the MHD ideal case.

## Introduction

Fluid turbulence is characterized by the phenomenon of local emergency of *Beltrami flows*, i.e. structures where velocity and vorticity field are strongly correlated, as long as nonlinear interactions take place, producing smaller and smaller scales^1–3^. This property of nonlinear interactions has been used as a basic ingredient in the formal proof of Onsager conjecture^4,5^, about the existence of weak solutions of Euler equations which do not conserve kinetic energy of the flow^6–8^. It is then worth investigating if a similar behaviour is found inside magnetohydrodynamic (MHD) incompressible turbulent flows, in the perspective to derive which mathematical properties characterize ideal turbulent flows, i,e. turbulent flows where dissipative terms tend to zero.

The formation of large scale correlated structures (*self-organization*), as the result of the relaxation processes occurring on very long times in MHD incompressible turbulent flows, has been the object of a lot of theoretical and numerical studies, which have shown that the properties of these structures could be predicted assuming that dissipative terms minimize the total energy of the turbulent flow, holding constant some ideal quadratic (rugged) invariants. In particular in 3D these invariants are cross helicity and magnetic helicity^9–16^, while in 2D they are cross helicity and magnetic potential^13,15,17–20^.

The properties of the coherent structures predicted by these studies have also been observed in solar wind data^10,18,21,22^.

Matthaeus *et al*.^23^ have shown that in both 2D and 3D incompressible MHD simulations dynamical alignment of velocity and magnetic field occurs on rapid times (of the order of some eddy turnover time)^24^.

The occurrence of coherent structures inside turbulence^15^ has also been discussed in relation to the phenomenon of spatial intermittency^2^. In particular, nonlinear interactions give rise to a cascade towards smaller lengths which is not self similar in that the Probability Distribution Functions of fluctuations at a given length display larger ad larger tails with respect to a Gaussian Distribution as the lengths become smaller and smaller. This behavior, which has been described as multifractal^25^, is now interpreted in terms of the occurrence at small scales of coherent structures, which are superposed on a background of random fluctuations^26^.

In this work we study in a systematic way, if and how phenomena similar to the local emergency of *Beltrami flows* in fluids occur on rapid ideal times in 2D MHD incompressible turbulent flows and how this eventual occurrence is related to the development of intermittency. In particular we want to assess if and how the ideal quadratic invariants play a role in determining the nature of the structures produced by nonlinear interactions by shaping these coherent structures in the very beginning of a decaying turbulence when dissipative terms have not yet developed their effects. To try to assess the general validity of the results obtained, our analysis has been performed for some different simulations, varying resolution, kinetic viscosity, which has always been put equal to magnetic diffusivity, and initial conditions.

The dynamical evolution of 2D incompressible MHD is described by the following set of equations

1 $$\begin{array}{rcl}\frac{\partial \omega }{\partial t}+{\bf{v}}\cdot \nabla \omega  & = & {\bf{B}}\cdot \nabla j+\nu {\nabla }^{2}\omega \\ \frac{\partial a}{\partial t}+{\bf{v}}\cdot \nabla a & = & \eta {\nabla }^{2}a\end{array}$$

where **v** and **B** are the fluid velocity and magnetic field which both have zero *z* component, *a*, *j* and *ω* are the z components of respectively the vector potential, the electric current density and the fluid vorticity and *ϕ* is the stream function such that $\omega =-{\nabla }^{2}{\varphi }$, while $\nu$ is the kinematic viscosity and *η* is the magnetic diffusivity^27,28^. Equations (1) are written in familiar Alfvén units^29^, with lengths scaled to *l*_0_, a typical large scale length. Velocity and magnetic field are scaled to the root-mean-square of the Alfvén speed *C*_*A*_ and time is scaled to ${l}_{0}/{C}_{A}$.

In their ideal form. i.e. when $\nu =\eta =0$, these equations conserve three global quadratic invariants (rugged invariants i.e. invariants which survive to any Galerkin truncation): the energy $E=\frac{1}{2}\int ({v}^{2}+{B}^{2}){d}^{2}r$, the cross-helicity ${H}_{c}=\frac{1}{2}\int {\bf{v}}\cdot {\bf{B}}\,{d}^{2}r$ and the mean square potential vector ${A}^{2}=\int {a}^{2}\,{d}^{2}r$.

In order to study the behavior of turbulent flows associated to these equations, numerical simulations with very low values of $\nu$ and *η* and periodic boundary conditions are usually performed. In such case, the long time evolution of these equations has been shown to give rise to the so-called *self-organization* of turbulent flows, in that large scale very correlated structures are finally obtained, whose properties can be derived from the idea that energy is minimized while holding constant cross-helicity and mean square magnetic potential^20^.

Let *λ* and *ϕ* be Lagrangian multipliers and Ω an open regular and limited domain of ${{\mathbb{R}}}^{2}$, imposing that

2 $$\delta [\frac{1}{2}{\int }_{{\rm{\Omega }}}({v}^{2}+{B}^{2}){d}^{2}r-\frac{\lambda }{2}{\int }_{{\rm{\Omega }}}{\bf{v}}\cdot {\bf{B}}\,{d}^{2}r-\varphi \,{\int }_{{\rm{\Omega }}}{a}^{2}{d}^{2}r]=0$$

and using the variational calculation analysis, we obtain the two relations

3 $$\begin{array}{rcl}{\bf{v}}-\lambda {\bf{B}} & = & 0\\ j-\lambda \omega -2{\varphi }\,a & = & 0\end{array}$$

which can be recast to the following form

4 $$\frac{\omega }{j}=\lambda ,\,\,\,\,\,\frac{j}{a}=\frac{1-{\lambda }^{2}}{2\varphi }={\phi }$$

where the presence of a correlation between *ω*, *j* and *j*, *a* is emphasized. In the present work we will explore the possibility that the above equilibria, which are intended to be long time solutions of equations (1), have an influence on the cascade processes, manifesting on time-scales comparable to an eddy turnover time.

## Results

### Simulations

System of equations (1) are solved in double periodic box, in a Cartesian geometry where each side is set to $2\pi {l}_{0}$. We compute the nonlinear terms using a pseudo-spectral technique, applying a 2/3 dealiasing rule^30,31^. The code conserves energy with high precision, and has been tested in absence of viscous/resistive terms (the spectral Galerking representations retain high accuracy and robustness, even in ideal MHD). A standard Laplacian dissipation with constant dissipation coefficients has been employed. The latter have been chosen high enough to guarantee the smoothness of the solutions, but also to achieve high Reynolds numbers. The values of the viscosity and the resistivity are reported in Table 1 together with a description of the runs performed. Time integration is achieved through a classical second-order Runge-Kutta method, which has been tested to be stable and robust, for each simulation. The number of used mesh points N^2^ goes from 2048^2^ *to* 4096^2^, and results have been found to be independent of this choice.

**Table 1:** Parameters for runs: *N* represents the number of grid points in each direction, $\nu$ and *η* are the viscosity and the magnetic diffusivity respectively, *M* is related to *N* through $M=lo{g}_{2}(N)$.

Runs	***N***	${\boldsymbol{\nu }}={\boldsymbol{\eta }}$	${\boldsymbol{M}}$
*RUN*1	2048	3 × 10^−4^	*M* = 11
*RUN*2	2048	2 × 10^−3^	*M* = 11
*RUN*3	4096	5 × 10^−4^	*M* = 12

Considering the representation of the magnetic and velocity fields in Fourier space, the energy is initially concentrated in the shell $1\le k\le 2$ *(* $k$ is the modulus of the wavenumber in units of $\mathrm{1/}{l}_{0}$). The initial energy has been normalized such that $E=\frac{1}{2}\langle {v}^{2}+{b}^{2}\rangle =1$, where the brackets denote a spatial average. Random phases are employed for the initial Fourier coefficients and uncorrelated, equipartitioned velocity and magnetic field fluctuations are imposed. This gives a negligible initial net cross helicity in the system. This choice of initial conditions corresponds in the physical space to a collection of energy containing magnetic island and vortical flows. The fields are therefore a superposition of large scale fluctuations, which suddenly undergo a state of fully developed turbulence. As we have just outlined, using the normalization described above, kinetic and magnetic Reynolds numbers of our simulations are respectively nothing but the inverse of the kinematic viscosity and magnetic diffusivity. We have finally considered an homogeneous spatial grid $({x}_{i},{y}_{k})$, such that ${x}_{i}=i\,\delta x$ and ${y}_{k}=k\,\delta y$, with $1\le i,j\le {2}^{M}$ and $\delta x=\delta y=\delta ={l}_{0}\,{2}^{-M}$ the mesh spacing.

### Analysis

**Figure 1:**
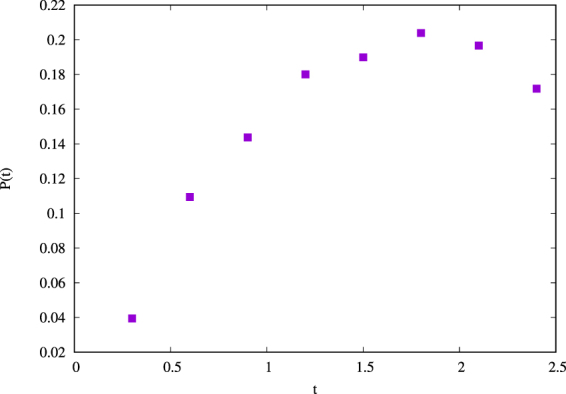
Dissipative power as a function of time, for *RUN*3. $P(t)=\eta  < {j}^{2}(t) > \,+\,\nu  < {\omega }^{2}(t) >$

**Figure 2:**
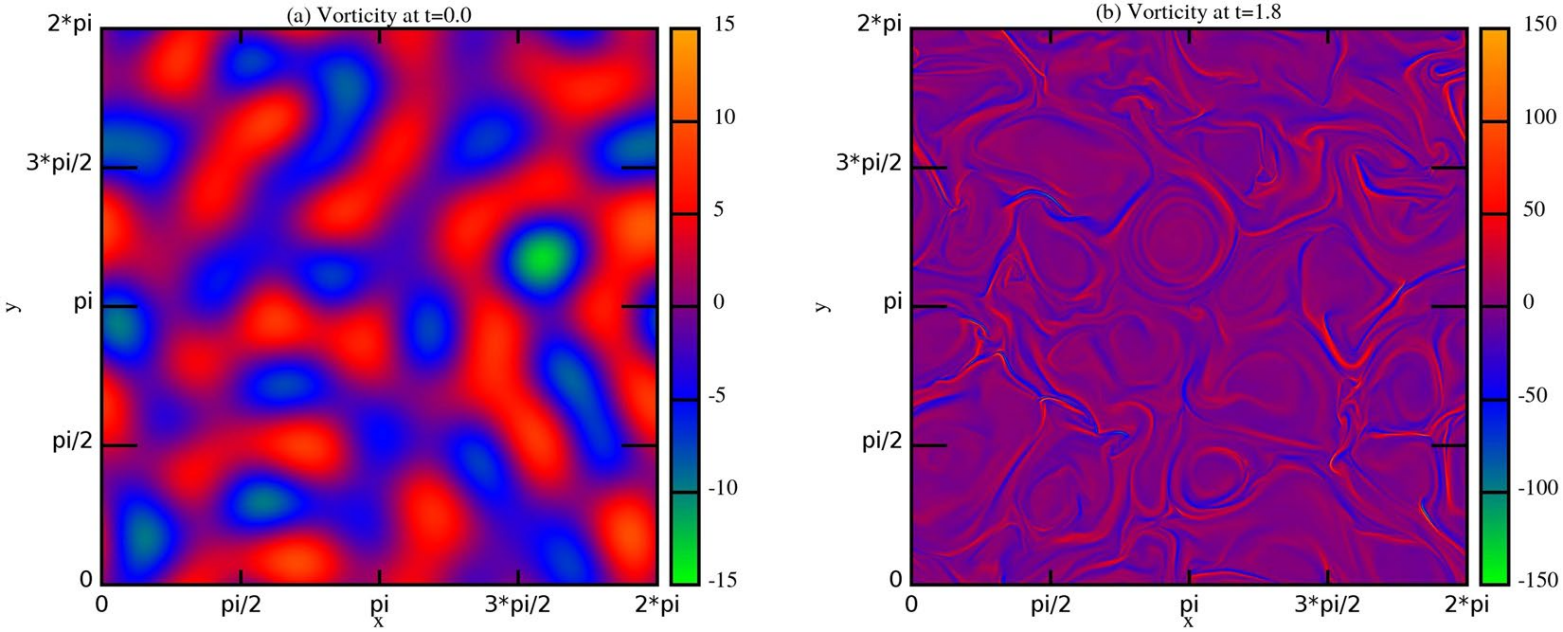
Contour plots of the vorticity field at times (**a**) $t=0.0$ and (**b**) $t=1.8$ for *RUN*3.

In order to identify the intermittent pattern formed at small scale in turbulent flow, we used a wavelet decomposition analysis, through an Haar wavelet base in two dimensions^26,32–38^. In accordance with the wavelet decomposition, for $1\le m\le M$, we define the scale ${l}_{m}={2}^{m-M}{l}_{0}={2}^{m}\delta$ (see section Methods). The wavelet decomposition has been performed on both the two components of velocity and magnetic field, choosing the fields at a simulation time where the turbulence was fully developed, a time ${t}^{\ast }$ which we have identified with the ideal time corresponding to the maximum value of the dissipated power (i.e. at this time the conditions of stationary state are valid since at the smallest scale the nonlinear effects are comparable with respect to the dissipative ones). For $RUN3$, for example, ${t}^{\ast }=1.8$ (Figure 1). It is worth noting that at this time the field structure of the solution is completely different from that one found at the the starting time, thus showing that the solution of the MHD equations at this time has no particular relation with the imposed initial condition (Figure 2).

**Figure 3:**
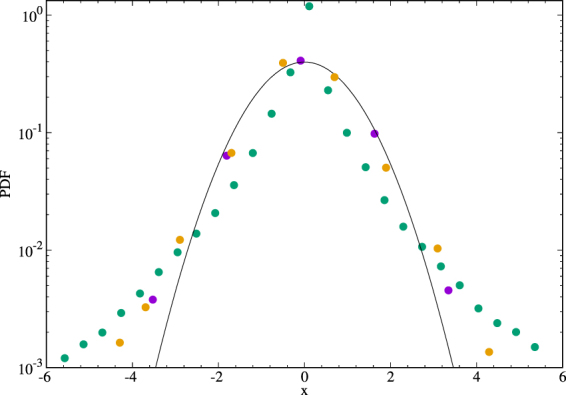
PDFs of ${{\rm{v}}}_{x}$ wavelet coefficients at scales ${l}_{1}$ , ${l}_{7}$  and ${l}_{8}$  at simulation time $t=1.8$ for *RUN*3, Gaussian Distribution $-$.

**Figure 4:**
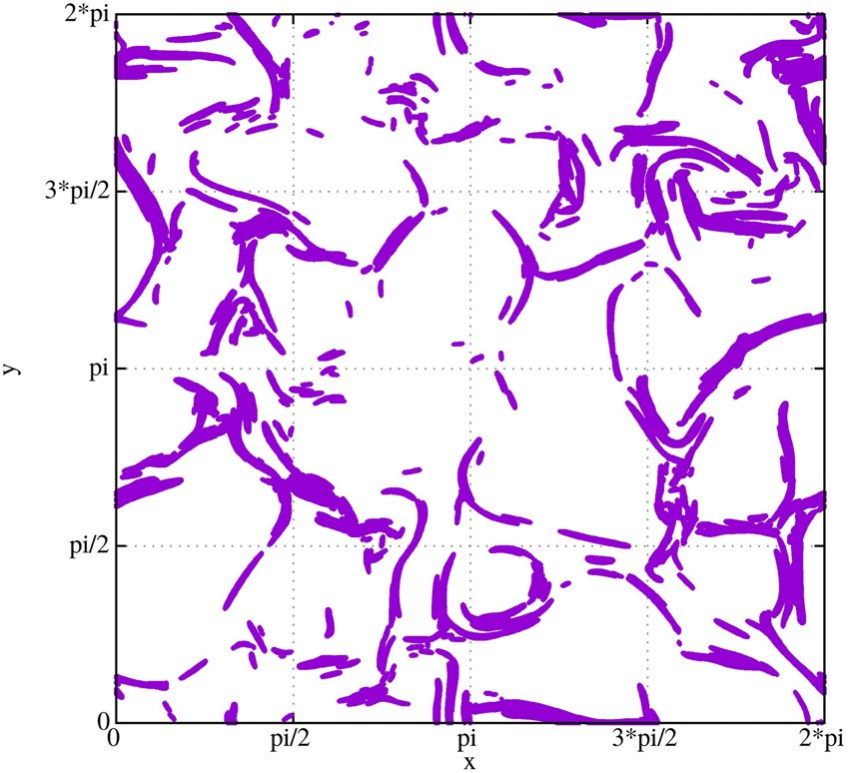
Grid points above-threshold at scale ${l}_{1}$ and time $t=1.8$ for $RUN3$.

As in an usual intermittent analysis^32^, we have then built up the PDFs of the velocity and magnetic wavelet coefficients at different scales. In Figure 3 we have represented the PDFs of the smallest scale (${l}_{1}$) and of the largest statistically significant scale (${l}_{8}$). Let us notice that, at the biggest considered scale (${l}_{8}$), the PDF is almost Gaussian, while, descending at smaller scales, important non-Gaussian tails are displayed. The standard deviation at each scale can now be used as a threshold for selecting the intermittent structures^32^. Actually we selected the wavelet coefficients of both the two components of the velocity and the magnetic field, whose module is greater than four times the corresponding field standard deviation. This operation allows the identification of the grid points, at fixed scale, where coherent intermittent structures are localised (Figure 4).

In order to study the properties of the correlation among the dynamical variables in the neighbor of each intermittent grid point $({x}_{i},{y}_{k})$ and for each scale ${l}_{m}$, we have considered such grid point as the center of a spatial window $[{x}_{i}-{{\rm{\Delta }}}_{m},{x}_{i}+{{\rm{\Delta }}}_{m}]\times [{y}_{k}-{{\rm{\Delta }}}_{m},{y}_{k}+{{\rm{\Delta }}}_{m}]$, with ${{\rm{\Delta }}}_{m}=2{l}_{m}$, we have then restricted the vorticity field, the current density field and the magnetic potential to each selected spatial window and we have hence calculated the correlation coefficient between the current density field and the vorticity field ${c}_{\omega ,j}^{m}({x}_{i},{y}_{k})$ and correlation coefficient between the current density field and the magnetic potential ${c}_{j,a}^{m}({x}_{i},{y}_{k})$ inside the above defined spatial windows.

**Figure 5:**
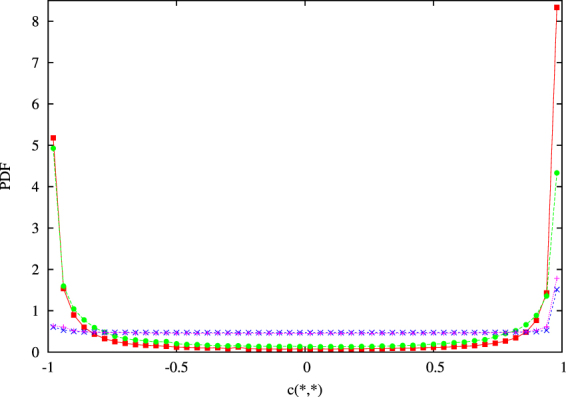
PDFs at scale ${l}_{1}$ of $\textcolor[rgb]{0,0,0}{\bullet }\,\,{c}_{\omega ,j}^{m}({x}_{i},{y}_{k})$, $\textcolor[rgb]{0,0,0}{\blacksquare}\,\,{c}_{j,a}^{m}({x}_{i},{y}_{k})$ at time $t=1.8$ calculated for the intermittent points and of $\textcolor[rgb]{0,0,0}{+}\,\,{c}_{\omega ,j}^{m}({x}_{i},{y}_{k})$, $\textcolor[rgb]{0,0,0}{\times }\,\,{c}_{j,a}^{m}({x}_{i},{y}_{k})$ at time $t=0.0$, calculated for the whole simulation domain, for $RUN3$.

In Figure 5 we present the PDF of ${c}_{\omega ,j}^{m}({x}_{i},{y}_{k})$ and ${c}_{j,a}^{m}({x}_{i},{y}_{k})$ calculated at $t=1.8$ in the neighbor of the intermittent structures, compared with the distribution of their initial values calculated on the whole simulation domain. Looking at these distributions, we find out that, in the simulation we are analyzing, the correlations between the current density field and the vorticity field and the current density field and the magnetic potential, which were almost null everywhere in the simulation domain at the initial time, are displaying after some ideal time well defined values in the neighbor of the small scale intermittent structures, thus showing that nonlinear interactions are extremely efficient in building up, rapidly in time and locally in the neighbor of the intermittent grid points, the correlations (4) predicted by the variational principle (2).

**Figure 6:**
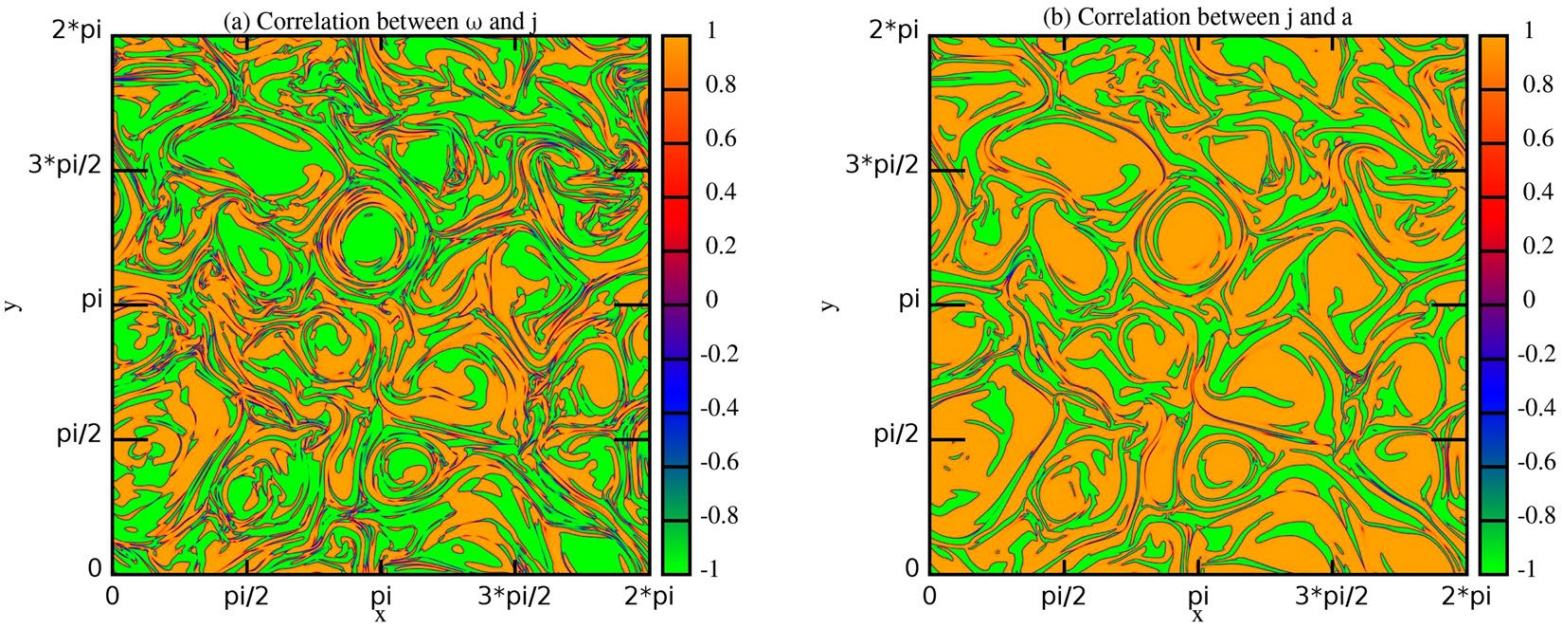
Contour plots of the correlation coefficients ${c}_{\omega ,j}^{m}({x}_{i},{y}_{k})$ (**a**) and ${c}_{j,a}^{m}({x}_{i},{y}_{k})$ (**b**) at scale ${l}_{1}$ and time $t=1.8$ for $RUN3$.

This result, very intriguing, has led us to repeat the same windowing analysis all over the simulation domain, i.e. for all grid points, and using the same previously used window of dimensions $(2{{\rm{\Delta }}}_{1}+1)\times (2{{\rm{\Delta }}}_{1}+1)$. Figure 6 displays the same pattern observed in Figure 4, clearly identifying large scale and intermittent structures, both characterized by strong correlations between $\omega$ and $j$ (Fig. 6(a)) and between $j$ and $a$ (Fig. 6(b)). Remarkably the sign of the correlations are opposite in the large scale regions with respect to that one of intermittent structures. Intermediate values of the correlations are present only in the very tiny regions where correlations change in sign, i.e. there is a passage from $-1$ to $+1$ or vice versa.

**Figure 7:**
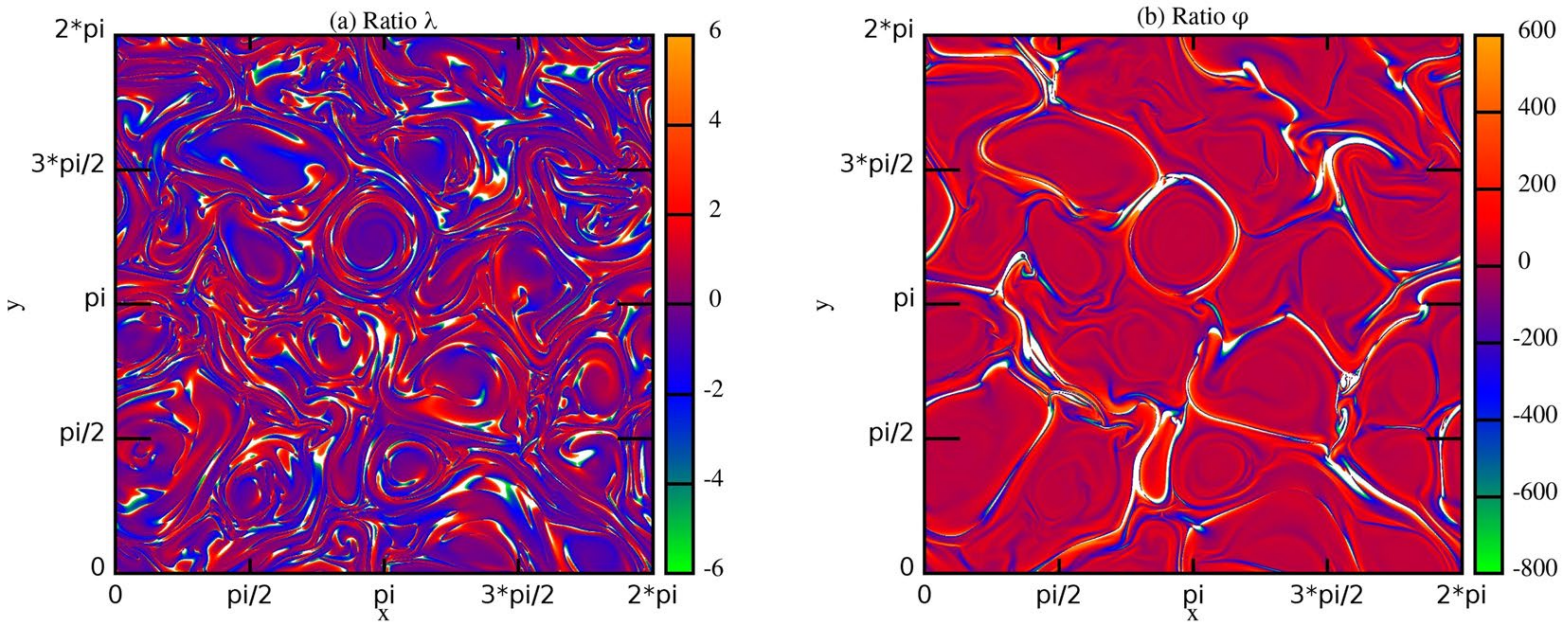
Contour plots of the ratios (4) at time $t=1.8$ for $RUN3$.

It is worth noting that looking at the contour plots of the $\lambda$-values and ${\phi }$-values given by relations (4) (Figure 7) the pattern obtained is practically superposed to that obtained in Figure 6. Large regions of space where the former values are almost constant can be identified, while in the intermittent regions the values of the ratios remain almost constant on the pattern identified by the previous intermittent analysis.

These results are particularly significant since they allow us to understand what kind of role the rugged invariant conservations play during the nonlinear evolution of the 2D MHD equations. In the initial condition the correlations between velocity and magnetic field and between current density and magnetic potential were present nowhere. Due to the effects of nonlinear interactions the correlations calculated over the whole simulation domain remain null, but now in the flow there are regions of maximum positive correlations and regions of maximum negative correlations and the separation between these regions correspond to a separation between large scale coherent structures and small scale intermittent structures. This means that the nonlinear interactions tend to segregate the structures with opposite sign of the correlations, not only in the physical space but also in the spectral space.

Starting from equations (1) for each grid point $({x}_{i},{y}_{k})$, we can define two typical evolution times for the variables $\omega$ and $a$. The first type is the time measured by an observer located in a fixed point of the space, it is defined for the vorticity field and the magnetic potential as

$${\mathop{t}\limits^{ \sim }}_{\omega }({x}_{i},{y}_{k})=\,\,|\frac{\omega ({x}_{i},{y}_{k})}{\frac{{\rm{\partial }}\omega }{{\rm{\partial }}t}({x}_{i},{y}_{k})}|,\,\,\,\,\,\,\,\,{\mathop{t}\limits^{ \sim }}_{a}({x}_{i},{y}_{k})=|\frac{a({x}_{i},{y}_{k})}{\frac{{\rm{\partial }}a}{{\rm{\partial }}t}({x}_{i},{y}_{k})}|$$

**Figure 8:**
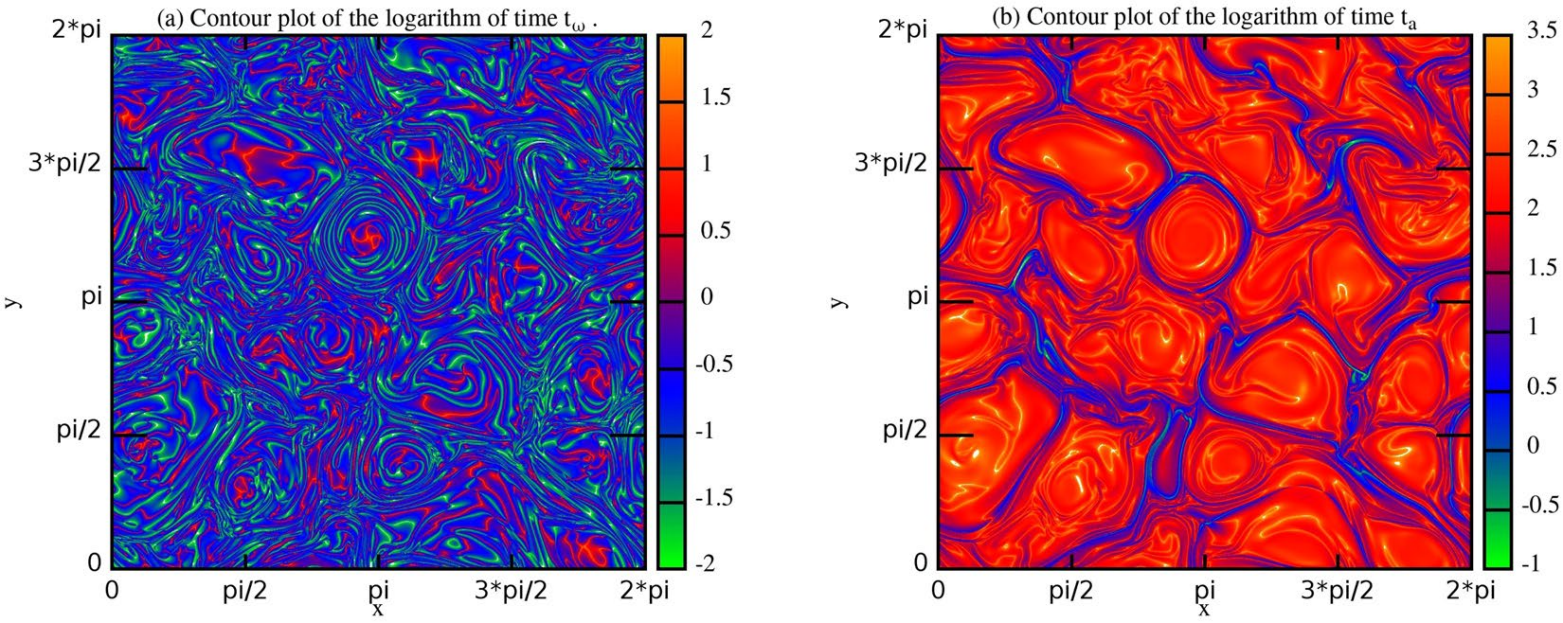
Contour plots of the characteristic times (5) at time $t=1.8$ for $RUN3$.

The other one is the time measured by an observer moving on a flux line, hence following the motion. In this case the characteristic times associated to the dynamical evolution of the vorticity field and the magnetic potential are

5 $${t}_{\omega }({x}_{i},{y}_{k})=|\frac{\omega ({x}_{i},{y}_{k})}{\frac{D\omega }{Dt}({x}_{i},{y}_{k})}|,\,\,\,\,\,\,\,\,{t}_{a}({x}_{i},{y}_{k})=|\frac{a({x}_{i},{y}_{k})}{\frac{Da}{Dt}({x}_{i},{y}_{k})}|$$

where $\frac{D\ast }{Dt}=\frac{\partial \ast }{\partial t}+v\cdot \nabla \ast$ is the material derivative. It is worth noting that in order to identify a typical life-time of the turbulent structures under our investigation, the latter time is much well suited that the former. In Figure 8 we have reported the contour plots of the life-times (5). The same pattern observed also in Figure 6, is displayed in Figure 8: large scale structures are characterized by long evolution time therefore they can be seen as quasi-steady structures, on the contrary, the intermittent structures have rapid evolution in time, associated to their dissipation. This result together with the localization of opposite values of the correlations in the intermittent and in the quasi-steady structures can furnish a key to understand why in decaying turbulence, on very long time, i.e. when small scale intermittent structures are finally dissipated, only one sign of the correlation at last survives on large scale structures (*self-organization*).

## Discussion

Using high resolution 2D MHD simulations we have analyzed and identified the rapid time formation of coherent structures induced by nonlinear interaction in MHD incompressible turbulent flows. These structures are characterized by the occurrence at small scales of regions where the correlations predicted by the hypothesis of quadratic rugged invariant conservations are locally present, a property which is the equivalent to the well known “Beltramization” of the fluid flows. Moreover large scale structures and intermittent structures present a clear separation also from the point of view of the calculated correlations, in that the two different types of structures are characterized by opposite signs of the correlations.

Also if a limited number of simulations have been performed, we think that the peculiar initial conditions we have chosen and the high resolution we have used, allow us to be sufficiently confident about the fact that the behavior observed and discussed represents a generic property of the 2D MHD turbulence. We have also tested other correlations derived from the variation principle where quantities which do not represent quadratic rugged invariants for the system were kept constant (for example the vorticity field and $f(a)={a}^{n}$ with $n > 2$). The obtained results have shown that these correlations are not locally present in the flow.

Analyzing the characteristic Lagrangian evolution times, other interesting properties of the coherent structures have emerged. Specifically, on very fast dynamic nonlinear time, local organization is observed; this is the case of intermittent structures, which are formed when the small scales are produced by the nonlinear interactions and are then dissipated. Conversely, on long evolution time, global self-organization is observed. In fact the large structures present really long evolution time, such that they can be considered quasi-steady structures. Consequently, leaving the simulation free to evolve, the rapid evolution finally destroys the intermittent structures, leaving only the large scale structures which are quasi-stable in time.

In ideal hydrodynamics the formation of singularities, i.e. the breakdown from smooth solutions, has necessary conditions given by the maximum norm of the vorticity (Beale-Kato-Majda theorem)^4,39^. A similar behavior is also observed for incompressible MHD equations: ideal MHD equations can be seen as the limit of the incompressible ones taking in consideration energy dissipation and magnetic helicity conservation (existence of a generalized Beale-Kato-Majda theorem)^40,28^. Therefore it is worth speculating that the intermittent small scale structures, observed in our simulations as consequence of the rapid time evolution produced by nonlinear interactions, could give rise to singular weak solutions when letting the dissipative coefficients go to zero. In this case the properties of these structures, identified by our analysis, could furnish a key to understand which mathematical conditions characterize singularity emergency in weak solutions of the MHD ideal case.

## Methods

For our analysis we have used a wavelet decomposition with an Haar basis. In the following we will furnish a brief description of this decomposition.

Let consider a two-dimensional $2\pi$-periodic function $f$ defined on a homogeneous spatial grid $({i}_{1},{i}_{2})$ with $1\le {i}_{1},{i}_{2}\le {2}^{M}$. Let also define for each $1\le m\le M$ the scale ${l}_{m}={2}^{m}\delta$, with $\delta$ the mesh spacing, such that fixed the scale ${l}_{m}$ we have ${2}^{M-m}$ grid points.

The two-dimensional orthonormal wavelet transform unfolds $f$ into scale, positions, and three directions using a function ${{\rm{\Psi }}}_{q}^{m}$, which is called two-dimensional mother wavelet

6 $${{\rm{\Psi }}}_{q}^{m}({\bf{i}}-{2}^{m}{\bf{k}})=\{\begin{array}{c}{\psi }_{m{k}_{1}}({i}_{1})\,{\psi }_{m{k}_{2}}({i}_{2}),\,\,q=1\\ {\psi }_{m{k}_{1}}({i}_{1})\,{\varphi }_{m{k}_{2}}({i}_{2}),\,\,q=2\\ {\varphi }_{m{k}_{1}}({i}_{1})\,{\psi }_{m{k}_{2}}({i}_{2}),\,\,q=3\end{array}\,{\rm{w}}{\rm{i}}{\rm{t}}{\rm{h}}\,{\bf{i}}=({i}_{1},{i}_{2}),\,{\bf{k}}=({k}_{1},{k}_{2})\,{\rm{a}}{\rm{n}}{\rm{d}}\,1\le {k}_{1},{k}_{2}\le {2}^{M-m},\,1\le m\le M$$

where ${\psi }_{m{k}_{\ast }}({i}_{\ast })$ is the one-dimensional mother wavelet and ${{\varphi }}_{m{k}_{\ast }}({i}_{\ast })$ is the one-dimensional scaling function. In our case these functions are defined as

7 $${\psi }_{mk}(i)={2}^{-\frac{m}{2}}\psi (\frac{(i-1)-{2}^{m}(k-1)}{{2}^{m}}),\,\,{\rm{w}}{\rm{h}}{\rm{e}}{\rm{r}}{\rm{e}}\,\,\,\psi (i)=\{\begin{array}{cc}1 & 0 < i\le \frac{1}{2}\\ -1 & \frac{1}{2} < i < 1\\ 0 & {\rm{o}}{\rm{t}}{\rm{h}}{\rm{e}}{\rm{r}}{\rm{w}}{\rm{i}}{\rm{s}}{\rm{e}}\end{array}\,{\rm{i}}{\rm{s}}\,{\rm{t}}{\rm{h}}{\rm{e}}\,{\rm{H}}{\rm{a}}{\rm{a}}{\rm{r}}\,{\rm{b}}{\rm{a}}{\rm{s}}{\rm{i}}{\rm{s}}\,{\rm{f}}{\rm{u}}{\rm{n}}{\rm{c}}{\rm{t}}{\rm{i}}{\rm{o}}{\rm{n}}$$

8 $${\varphi }_{mk}(i)={2}^{-\frac{m}{2}}\varphi (\frac{(i-1)-{2}^{m}(k-1)}{{2}^{m}}),\,\,\,{\rm{w}}{\rm{h}}{\rm{e}}{\rm{r}}{\rm{e}}\,\,\,\varphi (i)=\{\begin{array}{cc}1 & 0 < i < 1\\ 0 & {\rm{o}}{\rm{t}}{\rm{h}}{\rm{e}}{\rm{r}}{\rm{w}}{\rm{i}}{\rm{s}}{\rm{e}}\end{array}\,{\rm{i}}{\rm{s}}\,{\rm{t}}{\rm{h}}{\rm{e}}\,{\rm{s}}{\rm{c}}{\rm{a}}{\rm{l}}{\rm{i}}{\rm{n}}{\rm{g}}\,{\rm{f}}{\rm{u}}{\rm{n}}{\rm{c}}{\rm{t}}{\rm{i}}{\rm{o}}{\rm{n}}$$

These functions verify the following orthogonal conditions

9 $$\sum _{i=1}^{{2}^{M}}{\psi }_{mk}(i){\psi }_{nk^{\prime} }(i)={\delta }_{mn}{\delta }_{kk^{\prime} },\,\,\,\,\sum _{i=1}^{{2}^{M}}{{\varphi }}_{mk}(i){\psi }_{nk^{\prime} }(i)={\delta }_{mn}{\delta }_{kk^{\prime} }$$

The field $f$, having a mean value $\bar{f}$, can be decomposed into an orthogonal wavelet series

10 $$f({i}_{1},{i}_{2})=\bar{f}+\sum _{m=1}^{M}\,\sum _{q=1}^{3}\,\sum _{{k}_{1},{k}_{2}=1}^{{2}^{M-m}}{w}_{q}^{m}({k}_{1},{k}_{2}){{\rm{\Psi }}}_{q}^{m}({i}_{1}-{2}^{m}{k}_{1},{i}_{2}-{2}^{m}{k}_{2})$$

${w}_{q}^{m}$ are the wavelet coefficients given by ${w}_{q}^{m}= < f,{{\rm{\Psi }}}_{q}^{m} >$, where $< \ast ,\ast  >$ denotes the ${L}^{2}$-inner product defined by $< h,g > ={\int }_{{\rm{\Omega }}}h({\rm{x}})g({\rm{x}})d{\rm{x}}$. The coefficients measure fluctuations of $f$ around the scale ${l}_{m}$ and around positions **k**, along one of three directions $q$.

### Data availability

The datasets generated and analyzed during the current study are available from the corresponding author on reasonable request.

## References

[1] Moffatt HK (1985). Magnetostatic equilibria and analogous euler flows of arbitrarily complex topology. part 1. fundamentals. J. Fluids Mech..

[2] Frisch, U. *Turbulence: The Legacy of A. N. Kolmogorov* (Cambridge University Press, 1995).

[3] Farge M, Pellegrino G, Schneider K (2001). Coherent vortex extraction in 3d turbulent flows using orthogonal wavelets. Phys. Rev. Lett..

[4] Onsager L (1949). Statistical hydrodynamics. Il Nuovo Cimento Series.

[5] Constantin P, Majda A (1988). The beltrami spectrum for incompressible fluid flows. Comm. in Math. Phys..

[6] Nigro G, Carbone V (2015). Finite-time singularities and flow regularization in a hydromagnetic shell model at extreme magnetic prandtl numbers. New J. of Phys..

[7] De Lellis, C. & Székelyhidi, L. Jr. The euler equations as a differential inclusion. Ann. of Math. (2) **170**, 1417–1436 (2009).

[8] Buckmaster T, De Lellis C, Isett P, Székelyhidi L (2015). Anomalous dissipation for 1/5-hölder euler flows. Ann. of Math. (2).

[9] Meneguzzi M, Frisch U, Pouquet A (1981). Helical and nonhelical turbulent dynamos. Phys. Rev. Lett..

[10] Grappin R, Frisch U, Pouquet A, Leorat J (1982). Alfvenic fluctuations as asymptotic states of mhd turbulence. Astron. Astrophys..

[11] Grappin R, Leorat J, Pouquet A (1983). Dependence of mhd turbulence spectra on the velocity field-magnetic field correlation. Astron. Astrophys..

[12] Matthaeus, W. & Montgomery, D. *Statistical Physics and Chaos in Fusion Plasmas* (C. W. Horton, Jr. and L. E. Reichl, Wiley, New York, 1984).

[13] Gloaguen C, Leorat J, Pouquet A, Grappin R (1985). A scalar model for mhd turbulence. Physica D: Nonlinear Phenomena.

[14] Carbone V, Veltri P (1990). A shell model for anisotropic magnetohydrodynamic turbulence. Geophys. Astrophys. Fluid Dynamics.

[15] Veltri, P., Carbone, V., Lepreti, F. & Nigro, G. *Self-Organization in Magnetohydrodynamic Turbulence*, 8009–8028 (Springer New York, 2009).

[16] Stribling T, Matthaeus WH (1991). Relaxation processes in a low-order three-dimensional magnetohydrodynamics model. Physics of Fluids B: Plasma Physics.

[17] Grappin R (1986). Onset and decay of two-dimensional magnetohydrodynamic turbulence with velocity-magnetic field correlation. Phys. Fluids..

[18] Pouquet A, Meneguzzi M, Frisch U (1986). Growth of correlations in magnetohydrodynamic turbulence. Phys. Rev. A.

[19] Carbone V, Veltri P (1987). A simplified cascade model for m.h.d. turbulence. Astron. Astrophys..

[20] Ting A, Matthaeus WH, Montgomery D (1986). Turbulent relaxation processes in magnetohydrodynamics. The Physics of Fluids.

[21] Dobrowolny M, Mangeney A, Veltri P (1980). Fully developed anisotropic hydromagnetic turbulence in interplanetary space. Phys. Rev. Lett..

[22] Matthaeus WH, Goldstein ML, Montgomery DC (1983). Turbulent generation of outward-traveling interplanetary alfvénic fluctuations. Phys. Rev. Lett..

[23] Matthaeus WH, Pouquet A, Mininni PD, Dmitruk P, Breech B (2008). Rapid alignment of velocity and magnetic field in magnetohydrodynamic turbulence. Phys. Rev. Lett..

[24] Servidio S, Matthaeus WH, Dmitruk P (2008). Depression of nonlinearity in decaying isotropic mhd turbulence. Phys. Rev. Lett..

[25] Parisi, G. & Frisch, U. *On the singularity structure of fully developed turbulence*, 84–87 (North-Holland, 1985).

[26] Yoshimatsu K, Schneider K, Okamoto N, Kawahara Y, Farge M (2011). Intermittency and geometrical statistics of three-dimensional homogeneous magnetohydrodynamic turbulence: A wavelet viewpoint. Physics of Plasmas.

[27] Davidson, P. A. *Turbulence in Rotating, Stratified and Electrically Conducting Fluids* (Cambridge University Press, 2013).

[28] Biskamp, D. *Nonlinear Magnetohydrodynamics*. Cambridge Monographs on Plasma Physics (Cambridge University Press, 1997).

[29] Matthaeus WM, Lamkin SL (1986). Turbulent magnetic reconnection. The Physics of Fluids.

[30] Ghosh S, Hossain WM (1993). Matthaeus. The application of spectral methods in simulating compressible fluid and magnetofluid turbulence. Computer Physics Communications.

[31] Servidio S (2010). Statistics of magnetic reconnection in two-dimensional magnetohydrodynamic turbulence. Phys. of Plasma.

[32] Foufoula-Georgiou, E. & Kumar, P. *Wavelets in Geophysics*, vol. 4 (ACADEMIC PRESS, 1994).

[33] Meneveau C (1991). Analysis of turbulence in the orthonormal wavelet representation. J. of Fluid Mech.

[34] Farge M (1992). Wavelet transforms and their applications to turbulence. Ann. Rev. of Fluid Mech..

[35] Farge M, Schneider K, Kevlahan N (1999). Non-gaussianity and coherent vortex simulation for two-dimensional turbulence using an adaptive orthogonal wavelet basis. Physics of Fluids.

[36] Farge M, Schneider K, Devynck P (2006). Extraction of coherent bursts from turbulent edge plasma in magnetic fusion devices using orthogonal wavelets. Physics of Plasmas.

[37] Yoshimatsu Kea (2009). Wavelet-based coherent vorticity sheet and current sheet extraction from three-dimensional homogeneous magnetohydrodynamic turbulence. Physics of Plasmas.

[38] Okamoto N, Yoshimatsu K, Schneider K, Farge M (2014). Small-scale anisotropic intermittency in magnetohydrodynamic turbulence at low magnetic reynolds numbers. Phys. Rev. E.

[39] Beale JT, Kato T, Majda A (1984). Remarks on the breakdown of smooth solutions for the 3-d euler equations. Comm. in Math. Phys..

[40] Caflisch RE, Klapper I, Steele G (1997). Remarks on singularities, dimension and energy dissipation for ideal hydrodynamics and mhd. Communications in Mathematical Physics.

